# Controllability and observability in complex networks – the effect of connection types

**DOI:** 10.1038/s41598-017-00160-5

**Published:** 2017-03-10

**Authors:** Dániel Leitold, Ágnes Vathy-Fogarassy, János Abonyi

**Affiliations:** 1 Department of Computer Science and Systems Technology, University of Pannonia, Egyetem u. 10, H-8200 Veszprém, Hungary; 2 Department of Process Engineering, University of Pannonia, Egyetem u. 10, H-8200 Veszprém, Hungary; 3 Institute of Advanced Studies Kőszeg, Chernel u. 14, H-9730 Kőszeg, Hungary

## Abstract

Network theory based controllability and observability analysis have become widely used techniques. We realized that most applications are not related to dynamical systems, and mainly the physical topologies of the systems are analysed without deeper considerations. Here, we draw attention to the importance of dynamics inside and between state variables by adding functional relationship defined edges to the original topology. The resulting networks differ from physical topologies of the systems and describe more accurately the dynamics of the conservation of mass, momentum and energy. We define the typical connection types and highlight how the reinterpreted topologies change the number of the necessary sensors and actuators in benchmark networks widely studied in the literature. Additionally, we offer a workflow for network science-based dynamical system analysis, and we also introduce a method for generating the minimum number of necessary actuator and sensor points in the system.

## Introduction

Y. Y. Liu *et al*. started a new trend in network science when they become the first to analyse complex networks as dynamical systems with the maximum matching algorithm^1^. They considered nodes as state variables, interpreted networks as linear multivariable dynamical systems and studied the controllability and observability of these models^2^. Based on these principles Yan *et al*. analysed the required energy for controlling a system^3^, Ruths & Ruths determined control profiles for cluster networks^4^, Pósfai, Liu, Slotine & Barabási examined how the degree correlation influences the required inputs^5^, and the robustness of an input configuration was also improved by X. Liu *et al*.^6^. The application of the proposed method is also widespread, for example, Penn, Knight, Chalkias, Velenturf & Lloyd applied this approach on fuzzy cognitive maps as well^7^. These studies impressively show the benefits of network science-based analysis of dynamical systems.

Despite the groundbreaking successes, some critiques have also been received. Müller & Schuppert determined that in transcriptional networks the method drastically overestimates the number of necessary inputs^8^. Sun, Cornelius, Kath & Motter also highlighted that the methodology needs further clarification because the method gives incorrect results for non-linear systems even for small examples^9^. This fact has also been evinced by Dunne, Williams, & Martinez^10^. Another problem is that researchers examined the correlation between necessary inputs generated by the proposed method, and structural properties, like degree distribution, but they did not take into account that the result of the maximum matching algorithm is not unique^11^.

The most contestable point of the network-based analysis is that it is based on a static and structural view of the system. We wish to offer a solution to the previously mentioned problems by examining how system dynamics should be represented realistically. The usage of proper topology is important and a crucial part of network analysis, as this is the only way to emphasise dynamics in statical representations. We introduce connection types according to the typical relationships of the state variables. To analyse how the determined connection types influence the controllability and observability of dynamical systems we developed a MATLAB toolbox. We examined 35 example networks used in articles and found that 27 do not represent dynamical systems. By comparing them with 18 independently selected dynamical systems, we revealed significant differences. While in dynamical systems the number of inputs and outputs does not change when the proper topology of the model is studied, in the case of other networks more than 95% of inputs and outputs disappeared because of the determined connection types.

In the following section, after a brief introduction to the network science-based analysis of dynamical systems, we present how the functional relationships of state variables define different types of connections, and propose a workflow to evaluate how these connection types influence the number of necessary actuators and sensors that ensure controllability and observability. As result, we conclude that in most of the articles dealing with network science-based controllability and observability analysis mainly the physical topologies of the systems are studied, while the analysis of the realistic state-transition matrix-based topologies can lead to significantly different conclusions.

## Results

### Controllability and observability analysis of dynamical systems

Linear time-invariant systems are mostly represented by state-space models

1a $$\dot{{\boldsymbol{x}}}(t)={\bf{A}}{\boldsymbol{x}}(t)+{\bf{B}}{\boldsymbol{u}}(t),$$

1b $${\boldsymbol{y}}(t)={\bf{C}}{\boldsymbol{x}}(t)+{\bf{D}}{\boldsymbol{u}}(t),$$

where ***x*** stands for state variables, ***u*** represents the inputs, i.e. the actuators, and ***y*** is the vector of outputs, i.e. the sensors of the system. **A** and **B** matrices define how state variables and inputs influence the change in the state variables, while matrices **C** and **D** define how state variables and inputs influence the outputs, respectively. Assuming that the number of internal state variables is *N*, the number of inputs is *M* and the number of outputs is *K*, then the sizes of the matrices are ${\bf{A}}\in {\Re }^{N\times N}$, ${\bf{B}}\in {\Re }^{N\times M}$, ${\bf{C}}\in {\Re }^{K\times N}$, ${\bf{D}}\in {\Re }^{K\times M}$.

Controllability and observability are certainly the two most important properties of dynamical systems. A system is controllable if we can drive the state variables from any initial to any desired values within a finite period of time with properly selected inputs^12^. A system is observable if we can determine the state of the system based on the recorded input and measured output variables^12^.

To ensure controllability with a minimal number of inputs the brute force approach should generate 2^*N*^ − 1 configurations of the **B** matrix. To solve this challenging task, Y. Y. Liu *et al*. proposed the maximum matching algorithm based on the network representation of the **A** matrix to select the control^1^ and observer^2^ nodes that ensure controllable and observable systems.

### Network based representation and analysis of dynamical systems

**Figure 1:**
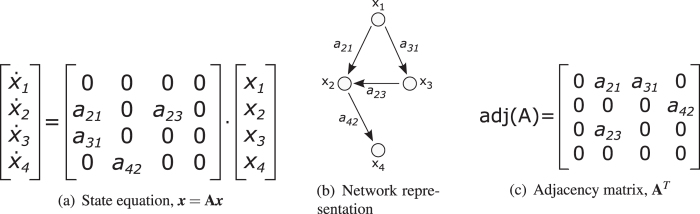
Representations of a state-transition matrix. (**a**) Illustrative state equation with four state variables. (**b**) Network representation of the dynamical system. (**c**) The adjacency matrix of the network is the transpose of the state-transition matrix.

Since the goal is to determine the inputs and outputs of a given system based on the structure of the state transition matrix, the network is defined based on matrix **A** (Fig. 1).

**Figure 2:**
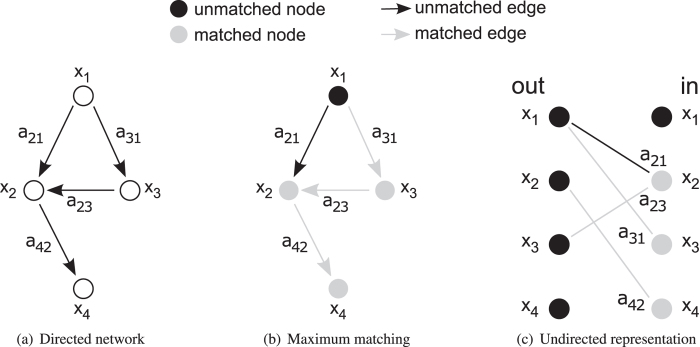
Maximum matching in directed networks. (**a**) Simple, unweighted directed graph with four nodes and four edges. (**b**) Result of maximum matching in the directed network. Edge *a*_21_ cannot be a member of the disjoint set of edges, as it has a common starting point with *a*_31_ and a common end point with *a*_23_. End points of matched edges are matched nodes. (**c**) Undirected representation of the directed network. For each node in the directed network there are two nodes in the undirected representation, one for outgoing edges and one for incoming edges. The representation of edges is obvious. The result of maximum matching is the same in both directed and undirected representations.

The maximum matching algorithm is a combinatorial method which creates the largest disjoint edge set in a graph. Although the maximum matching is interpreted on undirected graphs, it is interpretable in a directed network as well (Fig. 2)^1^. The disjoint condition in a directed network means that two edges cannot have a common starting point or common end point. A directed network can be represented as an undirected network as well (Fig. 2). The result of a matching is the disjoint edge set and its size, furthermore, matched and unmatched edges and nodes can be determined. An edge is matched if it is in the disjoint edge set, otherwise, it is unmatched. A node is matched, if it is an endpoint of a matched edge, otherwise it is unmatched. The result of maximum matching is a matching with maximum size. If a matching results in all nodes as matched nodes in the network, then it is called a perfect matching.

According to Y. Y. Liu *et al*., if we determine the unmatched nodes of the network, which are associated with matrix **A**, then we obtain the driver nodes^1^. Driver nodes are the nodes in the network that are influenced by the inputs. If we transpose the network, i.e. change the direction of edges, then the generated unmatched nodes are the sensor nodes of the same system. Similarly, sensor nodes are the nodes that are observed by outputs. With these driver and sensor nodes the system will be controllable and observable. The methodology provides only a structural analysis of a system, i.e. it deals with the structural architecture exclusively, and it ignores edge weights. The determined driver and sensor nodes are also just structural positions in the system, the methodology does not assign any parameters to matrices **B** and **C** or vectors ***u*** and ***y***. Since the maximum matching algorithm generally does not provide a unique solution, the provided driver and sensor nodes can be different for the same topology. The methodology accepts all of these solutions as a result and does not evaluate them according to other aspects.

Nevertheless, an exceptional case is known: with perfect matching the number of unmatched nodes is zero. In this case, one driver node grants the controllability independently from its location. To understand the mechanism of controlling, Liu *et al*. introduced the concept of stem and cycle^13^. A stem is a directed path in the network starting with a driver node. Cycles are controlled by the inputs, which have at least one node that has an incoming edge from a controlled node. A node is controlled, if it is a member of the stem or a controlled cycle. Perfect matching can occur for parts of the network as well. Namely, inauspicious cases cycles are controlled by stems or other cycles. Rarely new driver nodes have to be appointed in these subsystems. Thus, in these unique situations, controlling the unmatched nodes is not enough to grant controllability. So far, only one approach handle this problem, referred as the signal sharing method, handles this issue^2^. Unfortunately, initial maximum matching is not unique. Therefore, signal sharing can provide a different number of driver nodes for a given topology. To analyse the effects of dynamics in systems, another method, referred to as path finding method, was used and also recommended to provide the minimum number of driver nodes. The mechanism of both methods is introduced in Section II in Supplementary Information.

### Connections between the state variables and their effect on controllability and observability

The connection between two state variables is represented by the sub-matrix that belongs to nodes *i* and *j*:

2 $${{\bf{A}}}^{(i,j)}=[\begin{matrix}{a}_{ii} & {a}_{ij}\\ {a}_{ji} & {a}_{jj}\end{matrix}].$$

**Figure 3:**
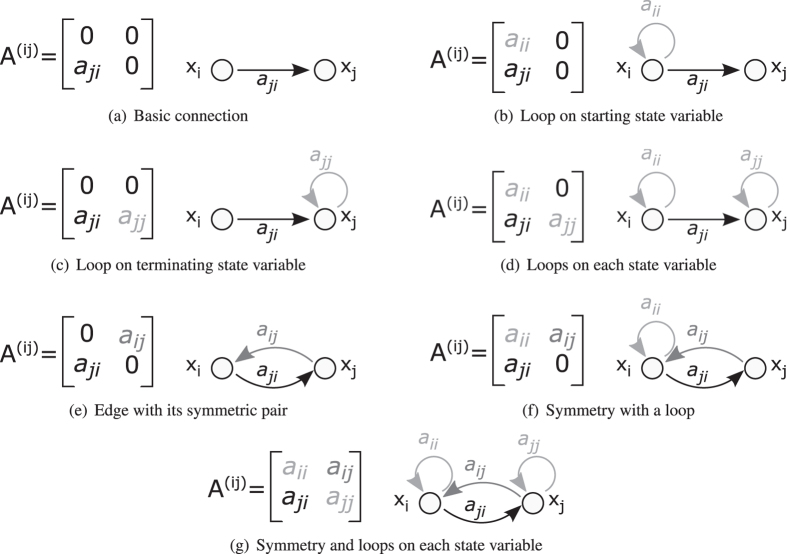
Dynamics of two state variables. (**a**) Basic connection without additional edges represents the ‘logical’ or ‘structural’ relationship between the elements. (**b**) Source state variable influences itself as well, for example, it represents a variable that has a capacity which changes due to the connection. (**c**) The change of the terminating state variable also depend on its value, e.g. accumulation. (**d**) Combined dynamics of types (**b**,**c**). (**e**) The symmetric edge-pair shows that the influence is undirected, or the strength of the influence depends on the other state variable, as the signal flows in causal bond graphs^16^. (**f**) Combined dynamics of types (**b**,**e**). (**g**) Combined dynamics of types (**d**,**e**).

When there is a connection between the *x*_*i*_ and *x*_*j*_ state variables, we can generate 2^3^-1 different combinations of the non-zero elements (edges) (see Fig. 3). Diagonal elements represent loops that describe a variable that has integrating characteristic defined as:

3 $$\frac{{\rm{d}}{x}_{i}}{{\rm{d}}t}=f({\boldsymbol{x}}),\,{x}_{i}\in {\boldsymbol{x}}.$$

By interpreting the principles of conservation of mass, energy or momentum, it can be realised that several connections should contain loops and symmetrical edges. In the case of symmetrical relationship the strength of the interaction (change of the state variable) is also a function of both the source and the sink variables:

4 $$\frac{{\rm{d}}{x}_{i}}{{\rm{d}}t}=f({\boldsymbol{x}}),\,{x}_{j}\in {\boldsymbol{x}}\wedge \frac{{\rm{d}}{x}_{j}}{{\rm{d}}t}=f({\boldsymbol{x}}^{\prime} ),\,{x}_{i}\in {\boldsymbol{x}}^{\prime} .$$

**Figure 4:**
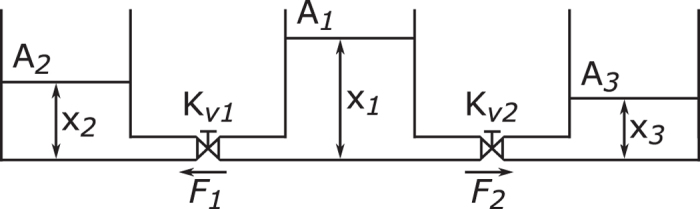
Physical representation of water tanks. State variables represent water levels in the tanks. Flow rates *F*_1_ and *F*_2_ show how the water flows through the pipes from the first tank into the others.

To illustrate connection types in a more tangible way, we illustrate them using a simple example. Let us consider three water tanks connected by two pipes (see Fig. 4).

**Figure 5:**
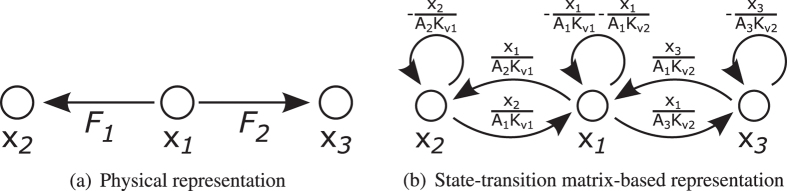
Physical representation and state-transition matrix-based representation of water tanks. The difference between the physical structure and the structure of the properly modelled state-transition matrix is significant.

The difference between physical topology and structure of the state-transition matrix (equation (5)) can be seen in Fig. 5. We supposed that the pressure/level is higher in tank 1 than in tank 2 or 3. Thus, the water can flow only in one direction, and the topology of the system remains unchanged. We use this supposition to ensure the linearity of the system. Although controllability of switching linear systems were examined, it is beyond the scope of this paper^14^.

5 $$[\begin{matrix}{\dot{x}}_{1}\\ {\dot{x}}_{2}\\ {\dot{x}}_{3}\end{matrix}]=[\begin{matrix}-\frac{1}{{A}_{1}{K}_{v1}}-\frac{1}{{A}_{1}{K}_{v2}} & \frac{1}{{A}_{1}{K}_{v1}} & \frac{1}{{A}_{1}{K}_{v2}}\\ \frac{1}{{A}_{2}{K}_{v1}} & -\frac{1}{{A}_{2}{K}_{v1}} & 0\\ \frac{1}{{A}_{3}{K}_{v2}} & 0 & -\frac{1}{{A}_{3}{K}_{v2}}\end{matrix}]\,[\begin{matrix}{x}_{1}\\ {x}_{2}\\ {x}_{3}\end{matrix}]$$

**Figure 6:**
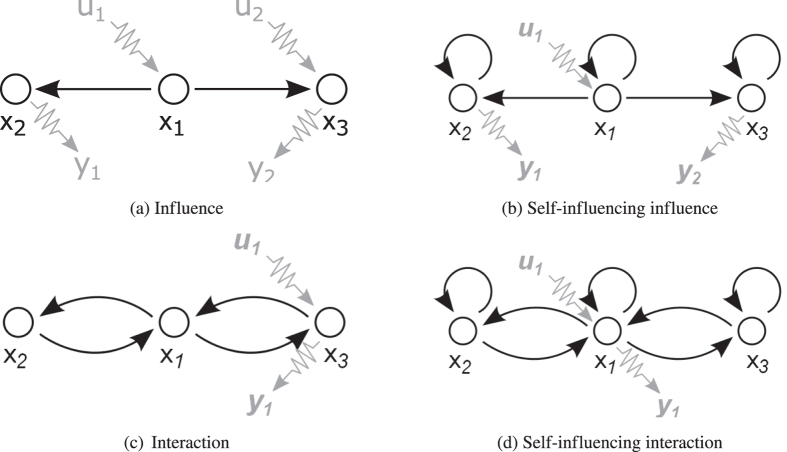
The examined four types of water-tank network models with associated inputs (***u***) and outputs (***y***). (**a**) Physical connection between state variables. (**b**) Self-influencing represents integrating node dynamics. (**c**) Interaction represents balanced dynamics (material balance). (**d**) Detailed model of the system. Already this small example clearly shows how connection types change the number and location of the driver and sensor nodes. The driver and sensor nodes were determined by the path-finding method introduced in section II of the Supplementary Information.

To analyse the effect of connection types, we defined four type of networks based on the four combinations of loops and edges. These four types are: networks that simply reflect the physical connections; networks with nodes having loops that represent the capacity or self-influencing of the state variables (the integrating behaviour of the tanks); networks with symmetric edge-pairs that reflect interactions between the state variables (mass balance); finally, networks with self-influence and interaction (Fig. 6). Notwithstanding, the effect of intrinsic nodal dynamics on the number of driver nodes was examined^15^, our approach differs, since it does not consider the different order of dynamics but the presence of first-order dynamics, therefore, the results also differ.

**Figure 7:**
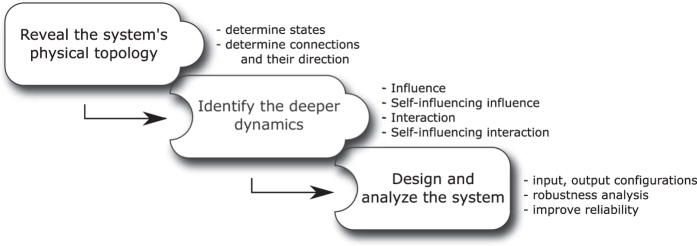
Determined flowchart for system design and analysis. Since connection types can be determined according to the topology of the systems, we strongly recommend following the proposed workflow and taking into account deeper dynamics to obtain more accurate and reliable results.

This example confirms that before generating input and output configurations the dynamical behaviour of the system must be examined more carefully, and the topology of the network has to be changed according to the required connection types. Figure 7 represents the flow-chart of this suggested workflow.

To extend the analysis of networks, a MATLAB toolbox called NOCAD (Network-based Observability and Controllability Analysis of Dynamical Systems) was implemented, which can examine the used topologies sophisticatedly. The toolbox is divided into three modules. The *network mapping* module creates a dynamical system from a network, i.e. an adjacency matrix is interpreted as a state-transition matrix, and **B**, **C** and **D** matrices of equation (1) are generated such that, the created linear system is controllable and observable. The *system characterization* is the second and the main module of the toolbox, and its task is to analyse the system and network specific measures. The *system investigation* module creates and analyses the modified topologies according to the proposed workflow.

### Used real networks

In the literature more or less the same set of networks is studied. The majority of them do not represent dynamical processes. Considering tools of system theory we established dynamics that are typical in dynamical systems, and with this knowledge we examined how these dynamics appear, and how they influence the properties of networks. To answer these questions we grouped networks into three subgroups according to their dynamics. Firstly, *Network Set I* contains networks which are examined in articles, and can represent dynamical processes. Topologies in this set usually originate from the field of regulatory, transcriptional, neuronal, power grid or watershed networks. In these networks dynamics are interpretable (in the sense of systems), i.e. some kind of capacity, or conservation law is observable between elements, such as Kirchhoff’s circuit laws, even if it does not appear in representations. Secondly, *Network Set II* contains such networks, where dynamics do not appear, e.g. in a social network or in an email network the information or the message can be propagated without limitations. Furthermore, the Internet, citation networks and food webs belong to this set also. It is interesting why dynamical systems and their state-transition matrices are not included in articles. Therefore, in *Network Set III* we included state-transition matrices of real dynamical systems, thus we were sure that results of these networks provide the real behaviour of dynamical systems. Sizes and short descriptions of networks can be found in Supplementary Table S1.

**Figure 8:**
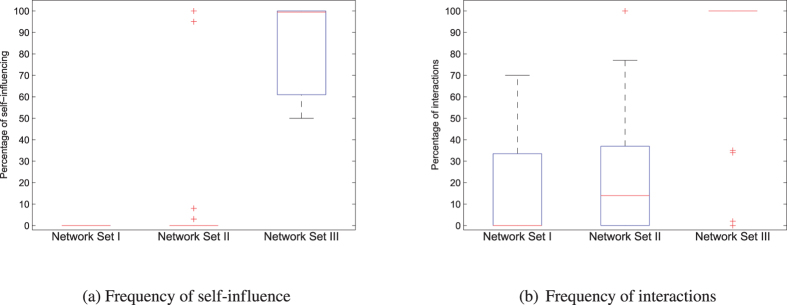
Initial percentage of self-influence and interactions in network sets. By examining real networks and dynamical systems, we get unequivocal results of structural differences. (**a**) Network Set I does not exhibit self-influence, and Network Set II does not exhibit them except in four cases, and only two of them exhibit more than 8% of self-influencing edges in their topologies. In contrast, more than 50% of self-influencing interactions in Network Set III are observed, i.e. more than half of the state variables influence themselves in dynamical systems. The median is almost 100%, so these dynamics are usual in dynamical systems. (**b**) In Network Set I usually less than 35% of interaction type edges are observed. In the case of Network Set II, results are a little higher. In Network Set III only four systems contain less than 100% of interactions, so in dynamical systems these dynamics is always present.

Firstly, to prove that the established connection types are important parts of dynamical systems, and are not of other networks, we analysed how many self-influences and interactions are in these topologies (Fig. 8). Results clearly show that these connection types are fundamental parts of dynamical systems, but rare in real networks. We concluded that this meaningful information about networks can be found in the literature, because only physical topologies were used to identify inputs and outputs instead of real state-space-based topology. Consequently, the determined number of driver and sensor nodes could be highly overestimated as is also shown by Müller & Schuppert^8^.

### Effect of connection types on controllability and observability

**Figure 9:**
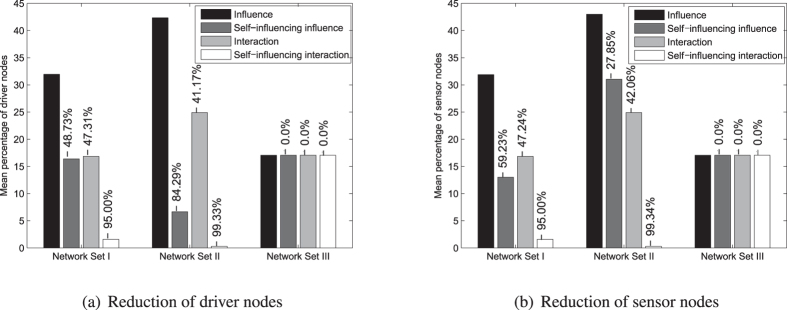
Proportion of driver and sensor nodes in networks with different connection types. Bar diagrams containing the mean of the proportion of driver (**a**) or sensor (**b**) nodes grouped according to network types. Apart from influence the numbers on the top of the bars show the reduction of driver and sensor nodes compared to the original.

The extension of the networks with new edges due to self-influences and interactions changes the required number of the driver and sensor nodes. The results can be seen in Fig. 9. It is an interesting fact that networks from Network Set I reacted differently in terms of node dynamics, i.e. exhibited self-influence influence, compared to networks from Network Set II. In more detail, if self-influence appears in networks from Network Set I, then they show more willingness to reduce sensor nodes, while in the case of Network Set II the reduction is rather significant in driver nodes.

Nevertheless, the more important result is that networks from Network Set III did not show any changes with the newly added edges, i.e. we can assume that the determined node and edge dynamics are parts of dynamical systems. In contrast, other examples exhibit more than 95% of driver and sensor reduction if both dynamics were taken into account. This drastic difference shows the importance of the presence of node and edge dynamics in topologies. In addition, we note that networks from Network Sets I and II could provide wrong results in this field if the researched area was sensitive to the lack of dynamics. Detailed results can be found in section IV of the Supplementary Information.

**Figure 10:**
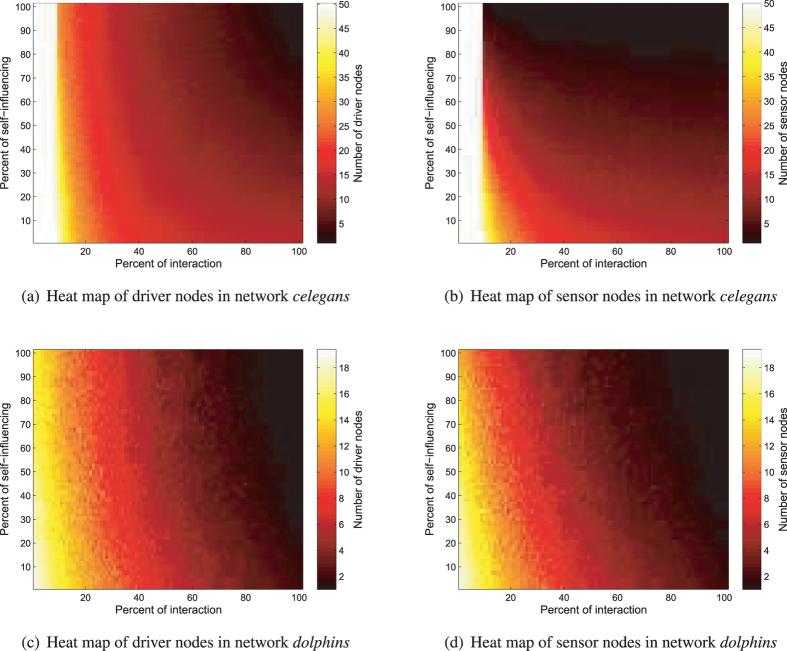
Number of driver and sensor nodes as a function of the presence of self-influence and interactions. The heat map clearly shows that with the increase in self-influencing and interaction type edges the number of driver and sensor nodes decreased progressively. The white bars in heat maps of *celegans* network are caused by the initial existence of 9% of interaction type connections.

The reduction of driver and sensor nodes can be addressed in terms of the newly created strongly connected components (SCC). A self-influencing edge creates an SCC that contains one node, while an interaction creates an SCC that contains two nodes, respectively. It is equal to one-length and two-length cycles, that can be controlled by a stem easily (for details, see section 2 in Supplementary Information). This phenomenon clearly shows, that the interpretation of self-influence on each node and the interpretation of interaction on each connected node-pair is not exaggerated: the number of driver or sensor nodes is progressively decreased by increasing the number of modified nodes and edges. To confirm our statement, we generated the heat map of network *celegans* from Network Set I and *dolphins* from Network Set II, that can be seen in Fig. 10. Furthermore, if both connection types appear in the network, then each connected component of a network can be controlled by one driver node and observed by one sensor node. Thus, with the help of Fig. 9 we can also conclude that networks from Network Set III contain more components than the others. As mentioned previously, the path-finding method was used to generate driver and sensor nodes. If we apply the signal sharing method, then the results are the same, only three cases differ slightly. In terms of influence, i.e. the original network, for Network Set I the method assigned 0.17605% more sensor nodes, while for Network Set II 0.00356% more driver and 0.010455% more sensor nodes were determined compared to the path finding method.

## Discussion

Although the network science-based examination of the system controllability and observability is a very popular and fruitful methodology, it is still in its infancy. Besides positive results, some negative comments were also published. Here, we provide answers to criticisms using the determined connection types. With a novel workflow, we illustrated how network-based analysis could generate more realistic results if the connections among the state variables would be defined based on more detailed analysis. We also realised that the majority of the networks studied in the literature do not exhibit dynamical behaviour that could be interpreted as a (linear) state space model. In the case of dynamical systems when the integrating behaviour and balance equation related relations among the state variables were also taken into account the number of necessary drivers and sensors decreased drastically compared to the analysis of oversimplified structural networks. For determining driver and sensor nodes a new approach was also recommended.

## Electronic supplementary material

Supplementary information

## References

[1] Liu Y-Y, Slotine J-J, Barabási A-L (2011). Controllability of complex networks. Nature.

[2] Liu Y-Y, Slotine J-J, Barabási A-L (2013). Observability of complex systems. Proceedings of the National Academy of Sciences.

[3] Yan G (2015). Spectrum of controlling and observing complex networks. Nature Physics.

[4] Ruths J, Ruths D (2014). Control profiles of complex networks. Science.

[5] Pósfai, M., Liu, Y.-Y., Slotine, J.-J. & Barabási, A.-L. Effect of correlations on network controllability. *Scientific Reports* **3** (2013). 10.1038/srep01067 PMC3545232 23323210

[6] Liu, X. *et al*. Minimum robust sensor placement for large scale linear time-invariant systems: a structured systems approach. In *4th IFAC Workshop on Distributed Estimation and Control in Networked Systems* (*NecSys*), 417–424 (2013).

[7] Penn, A. S., Knight, C. J., Chalkias, G., Velenturf, A. P. & Lloyd, D. J. Extending participatory fuzzy cognitive mapping with a control nodes methodology: a case study of the development bio-based economy in the humber region, uk. In Gray, S., Paolisso, M., Jordan, R. & Gray, S. (eds) *Environmental Modeling with Stakeholders* (Springer International Publishing, 2016).

[8] Müller F-J, Schuppert A (2011). Few inputs can reprogram biological networks. Nature.

[9] Sun, J., Cornelius, S. P., Kath, W. L. & Motter, A. E. Comment on “controllability of complex networks with nonlinear dynamics”. *arXiv preprint arXiv*:*1108*.*5739* (2011).

[10] Gates, A. J. & Rocha, L. M. Control of complex networks requires both structure and dynamics. *arXiv preprint arXiv*:*1509*.*08409* (2015). 10.1038/srep24456 PMC4834509 27087469

[11] Zhang X, Lv T, Yang X, Zhang B (2014). Structural controllability of complex networks based on preferential matching. PLOS ONE.

[12] Cameron, I. T. & Hangos, K. *Process modelling and model analysis*, vol. 4 (Academic Press, 2001).

[13] Liu Y-Y, Slotine J-J, Barabási A-L (2012). Control centrality and hierarchical structure in complex networks. PLOS ONE.

[14] Klamka, J. & Niezabitowski, M. Controllability of switched linear dynamical systems. In, *2013 18th International Conference on Methods and Models in Automation and Robotics* (*MMAR*), 464–467 (2013).

[15] Zhao, C., Wang, W.-X., Liu, Y.-Y. & Slotine, J.-J. Intrinsic dynamics induce global symmetry in network controllability. *Scientific Reports* **5** (2015). 10.1038/srep08422 PMC4325315 25672476

[16] Broenink, J. F. Introduction to physical systems modelling with bond graphs. *SiE Whitebook on Simulation Methodologies* 1–31 (1999).

