# Supplementary material for: Controllability and observability in complex networks – the effect of connection types
Source: Sci Rep. 2017 Mar 10;7:151. doi: 10.1038/s41598-017-00160-5 (PMC5427891; doi:10.1038/s41598-017-00160-5)
Supplement: Supplementary file 1 — Supplementary information [file 41598_2017_160_MOESM1_ESM.pdf]

# Supplementary Information: Controllability and observability in complex networks - the effect of connection types

Dániel Leitold[1], Ágnes Vathy-Fogarassy[1] and János Abonyi[*,2,3]

[1]Department of Computer Science and Systems Technology, University of Pannonia, Egyetem u. 10, H-8200 Veszprém, Hungary
[2]Department of Process Engineering, University of Pannonia, Egyetem u. 10, H-8200 Veszprém, Hungary
[3]Institute of Advanced Studies Kőszeg, Chernel u. 14, H-9730 Kőszeg, Hungary

January 2, 2017

[*]janos@abonyilab.com

# Contents

**I Introduction** **2**

**II The path-finding method** **2**

**III The studied networks** **4**

**IV Results** **6**

# I Introduction

The Supplementary Information is organized as follows: In Section II, we introduce our "path-finding" method which was used to calculate the necessary driver and sensor nodes. In Section III, we present the studied networks. In Section IV we show the results.

# II The path-finding method

The number of driver nodes can be defined as the sum of the number of unmatched nodes and the number of root strongly connected components (SCC), where all nodes are matched [1]. A root SCC, $R$, is a set of nodes, where there is no edge from node $x_j$ to node $x_i$ for all $x_j \notin R$ and for all $x_i \in R$. $R$ is a matched SCC if any node $x_i$ is matched by a node $x_j$ such that $x_i, x_j \in R$. Since a matched root SCC is inaccessible structurally, and uncontrolled as no unmatched node determined by maximum matching in it, we have to deal with this phenomenon separately. In the literature, one method deals with this problem using sharing input signals [1], but here, we recommend another method that can provides solution without the sharing of input signals and in some cases it grants input configuration with less driver nodes than provided by the existing method.

The signal sharing method achieves controllability by sharing the signal of an existing input on an arbitrary node from each matched root SCC. This is possible, since a matched root SCC can control itself, and only a shared signal necessary on one of its nodes. This is not true for unmatched nodes, we cannot control an unmatched nodes with a shared signal. Thus, the number of generated inputs is equal to the number of unmatched node, but the number of driver nodes is higher, it is increased by the number of matched root SCCs, since shared signals creates new driver nodes. The creation of a new approach was motivated by three reasons. The first is that we found that in some cases we can control a system with less driver nodes, than determined by the signal sharing method. The second was the presence of SCCs where unmatched nodes were identified, so we assumed that the phenomenon was not generated by SCC. The third was the fact that sharing an input signal in some applied area is impossible, and signal sharing makes controller design more complex. With the path-finding method we want to answer these remarks.

As a result of our research, we found that matched root SCCs are results of Hamiltonian cycles. To eliminate the sharing of an input signal, the method cuts each Hamiltonian cycle and creates a Hamiltonian path so that, if it is possible, then the path continues in an unmatched node. Formally, if the matched edge set is denoted by $M$, and matched root SCC by $R$, then we find nodes $x_1$, $x_2$, and $x_3$ such that $(x_2, x_1), (x_2, x_3) \in E$; $x_2, x_1 \in R, x_3 \notin R$ and $(x_2, x_1) \in M, (x_2, x_3) \notin M$. Then by removing $(x_2, x_1)$ from $M$ and by adding $(x_2, x_3)$ to $M$ we create a new maximum matching, where $x_1$ is an unmatched node in SCC $R$, and $x_3$ is a matched node. The signal sharing method determines $x_3$ as a driver node, and one node from $R$, while the path-finding method determines only $x_1$ a driver node. In Figure S1 the visualization of this example can be seen. If there is no such unmatched node, then the method only removes an edge from the matching, thus creates an unmatched node for all matched root SCCs. Since

our method identifies Hamiltonian paths, we called this new method *path-finding*, and the original one, as it shares signal, the *signal sharing* method.

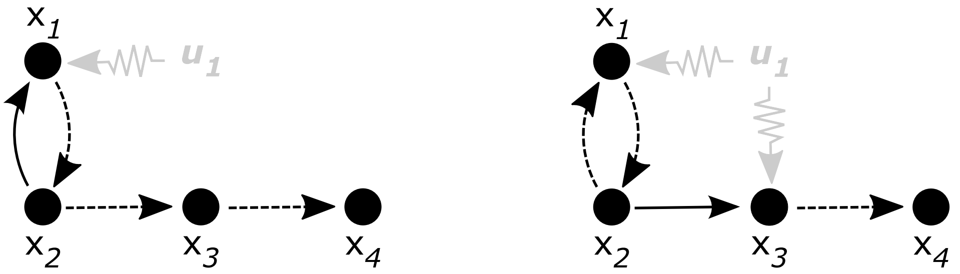


(a) Path finding method (b) Signal sharing method

Figure S1: **An illustrative example with input configurations ($\boldsymbol{u}$) generated by the path-finding and signal sharing methods.** Dashed lines show the matched edges. The unmatched node in (a) is $x_1$, and in (b) is $x_3$. In the case of the signal sharing method, node $x_3$ is controlled by $u_1$ and matched root SCC $\{x_1, x_2\}$ should be also controlled by this input, which results in another driver node: $x_1$. In contrast, the path-finding method modifies the maximum matching by the exchange of the edge $(x_2, x_1)$ with the edge $(x_2, x_3)$. By changing the edges, the path-finding method can control the system without sharing the input signal.

Although path-finding method simplifies the controlling process, the signal sharing method has its advantage as well. Since the path finding method assigns separate input for each matched root SCC, it can produce more inputs than signal sharing method, which can control all the matched root SCCs with only one input, as shown in Figure S2.

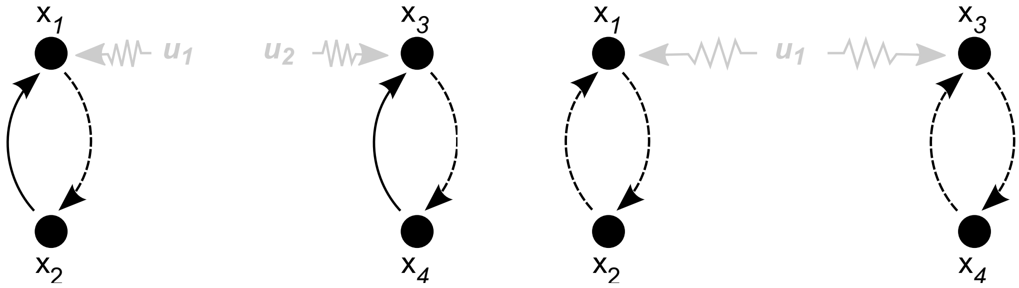


(a) Path finding method (b) Signal sharing method

Figure S2: **The input configurations ($\boldsymbol{u}$) for an unconnected system generated by the path finding and by signal sharing methods.** Dashed lines show the matched edges. It is clearly visible that the unconnected components increase the number of inputs in the case of the path-finding method. In contrast, the signal sharing method shares the signal of the only input, $u_1$.

The maximum matching method does not generate a unique solution, but matching of the same number of observer and controller nodes. The path finding method exhibits the same properties. The result of the path finding method is not unique, since it is possible that from a matched root SCC more unmatched nodes can be accessed. Here, the selection of Hamiltonian path determines the resultant input configuration. Nonetheless, the number of provided driver nodes is the same in each case for a given topology: the sum of the number of unmatched nodes and the number of those matched root SCCs, that do not point to any unmatched node. Here, we draw attention to a special case: if more than one matched root SCC point to an unmatched node, then only one matched root SCC can be eliminated from the sum.

In the case of the signal sharing method, the results can also be different, but the number of driver nodes depends on initial maximum matching (e.g. Figure S1).

## III The studied networks

The networks used in the article can be seen in Table S1. *Network Set I* contains networks, which are used in controllability examinations and their dynamical behaviour is interpretable. In contrast, topologies in *Network Set II* describe processes in which dynamical behaviours are not interpretable. *Network Set III* contains topologies which originate from state-transition matrices of real dynamical systems.

Most of the networks originated from different sources, as can be seen in Table S1, but almost all of the topologies of Network Set III are analysed in the same study. We need to mention that the state-transition matrices were available on the Internet [2], and they were revealed by the authors of [3], and the topologies were not created by us. We uploaded the data to our website also [4].

Table S1: **Topologies used in the study.** Network Set I contains 8 example networks, Network Set II contains 27 example networks, and Network Set III contains 18 example networks. The table shows the names, and the descriptions of the networks, and $N$ represents the number of nodes and $|E|$ stands for the number of edges.

| Type | Name | $N$ | $\lvert E\rvert$ | Description (Citation) |
|---|---|---|---|---|
| Network Set I | celegans | 297 | 2,345 | C elegans neural network. [5] |
| | ecoli | 418 | 519 | Transcriptional regulation network of E. coli. [6] |
| | mac71 | 71 | 746 | Macaque monkey visual cortex. [7] |
| | mac95 | 94 | 2,390 | Macaque monkey visual cortex. [8] |
| | s208 | 122 | 189 | Power grid network. [9] |
| | s420 | 252 | 399 | Power grid network. [9] |
| | s838 | 512 | 819 | Power grid network. [9] |
| | yeast | 688 | 1,079 | Protein-protein interaction network in yeast. [10] |
| Network Set II | amazon0302 | 262,111 | 1,234,877 | Amazon co-purchasing network, March 2 2003. [11] |
| | amazon0312 | 400,727 | 3,200,440 | Amazon co-purchasing network, March 12 2003. [11] |
| | amazon0505 | 410,236 | 3,356,824 | Amazon co-purchasing network, May 5 2003. [11] |
| | amazon0601 | 403,394 | 3,387,388 | Amazon co-purchasing network, June 1 2003. [12] |
| | berkstan | 26,475 | 106,762 | AS graph from RouteViews BGP table snapshots. [12] |
| | caida | 685,230 | 7,600,595 | Web graph of Berkeley and Stanford. [13] |
| | dolphins | 62 | 159 | Social network of bottlenose dolphins. [14] |
| | epinion | 75,879 | 508,837 | Who-trusts-whom network of Epinions.com. [15] |
| | freeman2 | 34 | 830 | Intra-organization network. [16] |
| | gnutella04 | 10,876 | 39,994 | Gnutella peer to peer network, August 4 2002. [17] |
| | gnutella05 | 8,846 | 31,839 | Gnutella peer to peer network, August 5 2002. [17] |
| | gnutella06 | 8,717 | 31,525 | Gnutella peer to peer network, August 6 2002. [17] |
| | gnutella08 | 6,301 | 20,777 | Gnutella peer to peer network, August 8 2002. [17] |
| | gnutella09 | 8,114 | 26,013 | Gnutella peer to peer network, August 9 2002. [18] |
| | gnutella24 | 26,518 | 65,369 | Gnutella peer to peer network, August 24 2002. [18] |
| | gnutella25 | 22,687 | 54,705 | Gnutella peer to peer network, August 25 2002. [18] |
| | gnutella30 | 36,682 | 88,328 | Gnutella peer to peer network, August 30 2002. [18] |
| | gnutella31 | 62,586 | 147,892 | Gnutella peer to peer network, August 31 2002. [18] |
| | google | 875,713 | 5,105,039 | Web graph from Google. [13] |
| | grass | 88 | 137 | Food web of Grassland. [19] |
| | notre | 325,729 | 1,497,134 | Web graph of Notre Dame. [20] |
| | pokec | 1,632,803 | 30,622,564 | Pokec online social network. [21] |
| | slash08 | 77,360 | 905,468 | Slashdot Zoo social network, November 6 2008. [13] |
| | slash09 | 82,168 | 948,464 | Slashdot Zoo social network, February 21 2009. [13] |
| | stanford | 281,903 | 2,312,497 | Web graph of Stanford.edu. [13] |
| | vote | 7,115 | 103,689 | Wikipedia who-votes-on-whom network. [22] |
| | ythan | 135 | 601 | Food web of Ythan Estuary. [19] |
| Network Set III | beam | 348 | 60,726 | Clamped beam model. [3] |
| | build | 48 | 1,176 | Motion problem in a building. [3] |
| | CDplayer | 120 | 240 | Classical CD player model. [3] |
| | eady | 598 | 357,406 | Model of an atmospheric storm track. [3] |
| | fom | 1,006 | 1,012 | Described in citepd article. [23] |
| | heatCont | 200 | 598 | Heat equation in a thin rod. [3] |
| | heatDisc | 200 | 598 | Discretization of the previous equation. [3] |
| | iss | 270 | 405 | Component 1r of the International Space Station. [3] |
| | MNA_1 | 578 | 1,694 | Modified Nodal Analysis model. [3] |
| | MNA_2 | 9,223 | 27,003 | Modified Nodal Analysis model. [3] |
| | MNA_3 | 4,863 | 13,921 | Modified Nodal Analysis model. [3] |
| | MNA_4 | 980 | 2,872 | Modified Nodal Analysis model. [3] |
| | MNA_5 | 10,913 | 54,159 | Modified Nodal Analysis model. [3] |
| | orrSom | 100 | 10,000 | Orr-Sommerfeld operator for Couette flow. [3] |
| | pde | 84 | 382 | Partial differential equation. [3] |
| | peec | 480 | 1,346 | Partial element equivalent circuit model. [3] |
| | random | 200 | 2,132 | Random example. [3] |
| | tline | 256 | 256 | Example of a transmission line model. [3] |

# IV Results

In Tables S2, S3, S4 and S5 the generated measures can be seen for the previously presented networks with Influence, Self-influencing influence, Interaction and Self-influencing interaction connection types, respectively.

Table S2: **Results related to the analysis of influence type connections.** $N$, the number of nodes, $|E|$ the number of edges, $D$ density, $< k >$ the average degree, $N_D$ the number of driver nodes, $N_S$ the number of sensor nodes, $\%_D$ the proportion of driver and all nodes, $\%_S$ the proportion of sensor and all nodes, $\%_{si}$ the percentage of self-influencing interactions, and $\%_{int}$ the percentage of interactions.

| Network | $N$ | $\|E\|$ | $D$ | $< k >$ | $N_D$ | $N_S$ | $\%_D$ | $\%_S$ | $\%_{si}$ | $\%_{int}$ |
|---|---|---|---|---|---|---|---|---|---|---|
| celegans | 297 | 2345 | 0.0266 | 15.7912 | 50 | 49 | 16.8350 | 16.4983 | 0.0000 | 9.1713 |
| ecoli | 418 | 519 | 0.0030 | 2.4833 | 314 | 314 | 75.1196 | 75.1196 | 0.0000 | 0.0000 |
| mac71 | 71 | 746 | 0.1480 | 21.0141 | 1 | 1 | 1.4085 | 1.4085 | 0.0000 | 70.3196 |
| mac95 | 94 | 2390 | 0.2705 | 50.8511 | 9 | 9 | 9.5745 | 9.5745 | 0.0000 | 57.7557 |
| s208 | 122 | 189 | 0.0127 | 3.0984 | 29 | 29 | 23.7705 | 23.7705 | 0.0000 | 0.0000 |
| s420 | 252 | 399 | 0.0063 | 3.1667 | 59 | 59 | 23.4127 | 23.4127 | 0.0000 | 0.0000 |
| s838 | 512 | 819 | 0.0031 | 3.1992 | 119 | 119 | 23.2422 | 23.2422 | 0.0000 | 0.0000 |
| yeast | 688 | 1079 | 0.0023 | 3.1366 | 565 | 565 | 82.1221 | 82.1221 | 0.0000 | 0.0927 |
| amazon0302 | 262111 | 1234877 | 0.0000 | 9.4225 | 8458 | 9238 | 3.2269 | 3.5245 | 0.0000 | 37.2403 |
| amazon0312 | 400727 | 3200440 | 0.0000 | 15.9732 | 14103 | 14382 | 3.5194 | 3.5890 | 0.0000 | 36.1965 |
| amazon0505 | 410236 | 3356824 | 0.0000 | 16.3653 | 14840 | 15062 | 3.6174 | 3.6715 | 0.0000 | 37.6065 |
| amazon0601 | 403394 | 3387388 | 0.0000 | 16.7944 | 8294 | 8398 | 2.0561 | 2.0818 | 0.0000 | 38.6337 |
| berkstan | 685230 | 7600595 | 0.0000 | 22.1841 | 261570 | 261687 | 38.1726 | 38.1897 | 0.0000 | 14.3038 |
| caida | 26475 | 106762 | 0.0002 | 8.0651 | 19112 | 19112 | 72.1889 | 72.1889 | 0.0000 | 100.0000 |
| dolphins | 62 | 159 | 0.0414 | 5.1290 | 19 | 19 | 30.6452 | 30.6452 | 0.0000 | 0.0000 |
| epinion | 75879 | 508837 | 0.0001 | 13.4118 | 41889 | 41792 | 55.2050 | 55.0772 | 0.0000 | 25.4096 |
| freeman2 | 34 | 830 | 0.7180 | 48.8235 | 1 | 1 | 2.9412 | 2.9412 | 0.0000 | 75.1055 |
| gnutella04 | 10876 | 39994 | 0.0003 | 7.3545 | 6004 | 6004 | 55.2041 | 55.2041 | 0.0000 | 0.0000 |
| gnutella05 | 8846 | 31839 | 0.0004 | 7.1985 | 5111 | 5111 | 57.7775 | 57.7775 | 0.0000 | 0.0000 |
| gnutella06 | 8717 | 31525 | 0.0004 | 7.2330 | 5033 | 5033 | 57.7378 | 57.7378 | 0.0000 | 0.0000 |
| gnutella08 | 6301 | 20777 | 0.0005 | 6.5948 | 4106 | 4106 | 65.1643 | 65.1643 | 0.0000 | 0.0000 |
| gnutella09 | 8114 | 26013 | 0.0004 | 6.4119 | 5355 | 5355 | 65.9970 | 65.9970 | 0.0000 | 0.0000 |
| gnutella24 | 26518 | 65369 | 0.0001 | 4.9302 | 18965 | 18965 | 71.5175 | 71.5175 | 0.0000 | 0.0000 |
| gnutella25 | 22687 | 54705 | 0.0001 | 4.8226 | 16478 | 16478 | 72.6319 | 72.6319 | 0.0000 | 0.0000 |
| gnutella30 | 36682 | 88328 | 0.0001 | 4.8159 | 26965 | 26965 | 73.5102 | 73.5102 | 0.0000 | 0.0000 |
| gnutella31 | 62586 | 147892 | 0.0000 | 4.7260 | 46227 | 46227 | 73.8616 | 73.8616 | 0.0000 | 0.0000 |
| google | 875713 | 5105039 | 0.0000 | 11.6592 | 423003 | 423964 | 48.3038 | 48.4136 | 0.0000 | 18.1161 |
| grass | 88 | 137 | 0.0177 | 3.1136 | 46 | 46 | 52.2727 | 52.2727 | 0.0000 | 0.0000 |
| notre | 325729 | 1497134 | 0.0000 | 9.0239 | 220552 | 221769 | 67.7103 | 68.0839 | 8.4288 | 34.8196 |
| pokec | 1632803 | 30622564 | 0.0000 | 37.5092 | 252075 | 252195 | 15.4382 | 15.4455 | 0.0000 | 37.3088 |
| slash08 | 77360 | 905468 | 0.0002 | 21.4106 | 49 | 6698 | 0.0633 | 8.6582 | 99.9315 | 76.5124 |
| slash09 | 82168 | 948464 | 0.0001 | 21.1800 | 3737 | 10495 | 4.5480 | 12.7726 | 95.2962 | 72.5722 |
| stanford | 281903 | 2312497 | 0.0000 | 16.4063 | 90168 | 90211 | 31.9855 | 32.0007 | 0.0000 | 16.0522 |
| vote | 7115 | 103689 | 0.0020 | 29.1466 | 4736 | 4736 | 66.5636 | 66.5636 | 0.0000 | 2.9049 |
| ythan | 135 | 601 | 0.0330 | 8.8444 | 69 | 69 | 51.1111 | 51.1111 | 2.9630 | 0.1678 |
| beam | 348 | 60726 | 0.5014 | 348.0000 | 1 | 1 | 0.2874 | 0.2874 | 50.0000 | 33.5893 |
| build | 48 | 1176 | 0.5104 | 48.0000 | 1 | 1 | 2.0833 | 2.0833 | 50.0000 | 35.2113 |
| CDplayer | 120 | 240 | 0.0167 | 2.0000 | 60 | 60 | 50.0000 | 50.0000 | 100.0000 | 100.0000 |
| eady | 598 | 357406 | 0.9994 | 1193.3378 | 1 | 1 | 0.1672 | 0.1672 | 100.0000 | 99.8924 |
| fom | 1006 | 1012 | 0.0010 | 0.0119 | 1003 | 1003 | 99.7018 | 99.7018 | 100.0000 | 100.0000 |
| heatCont | 200 | 598 | 0.0150 | 3.9800 | 1 | 1 | 0.5000 | 0.5000 | 100.0000 | 100.0000 |
| heatDisc | 200 | 598 | 0.0150 | 3.9800 | 1 | 1 | 0.5000 | 0.5000 | 100.0000 | 100.0000 |
| iss | 270 | 405 | 0.0056 | 2.0000 | 135 | 135 | 50.0000 | 50.0000 | 50.0000 | 100.0000 |
| MNA_1 | 578 | 1694 | 0.0051 | 4.6713 | 3 | 3 | 0.5190 | 0.5190 | 59.5156 | 100.0000 |
| MNA_2 | 9223 | 27003 | 0.0003 | 4.6358 | 4 | 4 | 0.0434 | 0.0434 | 60.9888 | 100.0000 |
| MNA_3 | 4863 | 13921 | 0.0006 | 4.5116 | 9 | 9 | 0.1851 | 0.1851 | 60.6827 | 100.0000 |
| MNA_4 | 980 | 2872 | 0.0030 | 4.6449 | 2 | 2 | 0.2041 | 0.2041 | 60.8163 | 100.0000 |
| MNA_5 | 10913 | 54159 | 0.0005 | 7.9417 | 10 | 10 | 0.0916 | 0.0916 | 99.1936 | 100.0000 |
| orrSom | 100 | 10000 | 1.0000 | 198.0000 | 1 | 1 | 1.0000 | 1.0000 | 100.0000 | 100.0000 |
| pde | 84 | 382 | 0.0541 | 7.0952 | 1 | 1 | 1.1905 | 1.1905 | 100.0000 | 100.0000 |
| peec | 480 | 1346 | 0.0058 | 4.3417 | 1 | 1 | 0.2083 | 0.2083 | 63.3333 | 100.0000 |
| random | 200 | 2132 | 0.0533 | 19.3200 | 1 | 1 | 0.5000 | 0.5000 | 100.0000 | 2.1682 |
| tline | 256 | 256 | 0.0039 | 0.0000 | 256 | 256 | 100.0000 | 100.0000 | 100.0000 | 0.0000 |

Table S3: **Results of examinations of networks with self-influence type connections.** $N$ the number of nodes, $|E|$ the number of edges, $D$ the density, $< k >$ the average degree, $N_D$ the number of driver nodes, $N_S$ the number of sensor nodes, $\%_D$ the proportion of driver and all nodes, $\%_S$ the proportion of sensor and all nodes, $\%_{si}$ the percentage of self-influencing connections, and $\%_{int}$ the percentage of interactions.

| Network | $N$ | $\|E\|$ | $D$ | $< k >$ | $N_D$ | $N_S$ | $\%_D$ | $\%_S$ | $\%_{si}$ | $\%_{int}$ |
|---|---|---|---|---|---|---|---|---|---|---|
| celegans | 297 | 2642 | 0.0300 | 15.7912 | 28 | 3 | 9.4276 | 1.0101 | 100.0000 | 9.1713 |
| ecoli | 418 | 937 | 0.0054 | 2.4833 | 312 | 76 | 74.6411 | 18.1818 | 100.0000 | 0.0000 |
| mac71 | 71 | 817 | 0.1621 | 21.0141 | 1 | 1 | 1.4085 | 1.4085 | 100.0000 | 70.3196 |
| mac95 | 94 | 2484 | 0.2811 | 50.8511 | 9 | 1 | 9.5745 | 1.0638 | 100.0000 | 57.7557 |
| s208 | 122 | 311 | 0.0209 | 3.0984 | 10 | 1 | 8.1967 | 0.8197 | 100.0000 | 0.0000 |
| s420 | 252 | 651 | 0.0103 | 3.1667 | 18 | 1 | 7.1429 | 0.3968 | 100.0000 | 0.0000 |
| s838 | 512 | 1331 | 0.0051 | 3.1992 | 34 | 1 | 6.6406 | 0.1953 | 100.0000 | 0.0000 |
| yeast | 688 | 1767 | 0.0037 | 3.1366 | 96 | 557 | 13.9535 | 80.9593 | 100.0000 | 0.0927 |
| amazon0302 | 262111 | 1496988 | 0.0000 | 9.4225 | 1 | 5668 | 0.0004 | 2.1624 | 100.0000 | 37.2403 |
| amazon0312 | 400727 | 3601167 | 0.0000 | 15.9732 | 1 | 12705 | 0.0002 | 3.1705 | 100.0000 | 36.1965 |
| amazon0505 | 410236 | 3767060 | 0.0000 | 16.3653 | 2 | 13712 | 0.0005 | 3.3425 | 100.0000 | 37.6065 |
| amazon0601 | 403394 | 3790782 | 0.0000 | 16.7944 | 155 | 1260 | 0.0384 | 0.3123 | 100.0000 | 38.6337 |
| berkstan | 685230 | 8285825 | 0.0000 | 22.1841 | 70171 | 8135 | 10.2405 | 1.1872 | 100.0000 | 14.3038 |
| caida | 26475 | 133237 | 0.0002 | 8.0651 | 1 | 1 | 0.0038 | 0.0038 | 100.0000 | 100.0000 |
| dolphins | 62 | 221 | 0.0575 | 5.1290 | 15 | 12 | 24.1935 | 19.3548 | 100.0000 | 0.0000 |
| epinion | 75879 | 584716 | 0.0001 | 13.4118 | 24377 | 15868 | 32.1261 | 20.9122 | 100.0000 | 25.4096 |
| freeman2 | 34 | 864 | 0.7474 | 48.8235 | 1 | 1 | 2.9412 | 2.9412 | 100.0000 | 75.1055 |
| gnutella04 | 10876 | 50870 | 0.0004 | 7.3545 | 20 | 5941 | 0.1839 | 54.6249 | 100.0000 | 0.0000 |
| gnutella05 | 8846 | 40685 | 0.0005 | 7.1985 | 118 | 4996 | 1.3339 | 56.4775 | 100.0000 | 0.0000 |
| gnutella06 | 8717 | 40242 | 0.0005 | 7.2330 | 79 | 4978 | 0.9063 | 57.1068 | 100.0000 | 0.0000 |
| gnutella08 | 6301 | 27078 | 0.0007 | 6.5948 | 80 | 3836 | 1.2696 | 60.8792 | 100.0000 | 0.0000 |
| gnutella09 | 8114 | 34127 | 0.0005 | 6.4119 | 76 | 5059 | 0.9367 | 62.3490 | 100.0000 | 0.0000 |
| gnutella24 | 26518 | 91887 | 0.0001 | 4.9302 | 331 | 18948 | 1.2482 | 71.4534 | 100.0000 | 0.0000 |
| gnutella25 | 22687 | 77392 | 0.0002 | 4.8226 | 335 | 16466 | 1.4766 | 72.5790 | 100.0000 | 0.0000 |
| gnutella30 | 36682 | 125010 | 0.0001 | 4.8159 | 229 | 26960 | 0.6243 | 73.4965 | 100.0000 | 0.0000 |
| gnutella31 | 62586 | 210478 | 0.0001 | 4.7260 | 303 | 46199 | 0.4841 | 73.8168 | 100.0000 | 0.0000 |
| google | 875713 | 5980752 | 0.0000 | 11.6592 | 162465 | 141104 | 18.5523 | 16.1130 | 100.0000 | 18.1161 |
| grass | 88 | 225 | 0.0291 | 3.1136 | 1 | 35 | 1.1364 | 39.7727 | 100.0000 | 0.0000 |
| notre | 325729 | 1795408 | 0.0000 | 9.0239 | 1 | 189150 | 0.0003 | 58.0697 | 100.0000 | 34.8196 |
| pokec | 1632803 | 32255367 | 0.0000 | 37.5092 | 114165 | 201024 | 6.9920 | 12.3116 | 100.0000 | 37.3088 |
| slash08 | 77360 | 905521 | 0.0002 | 21.4106 | 1 | 6698 | 0.0013 | 8.6582 | 100.0000 | 76.5124 |
| slash09 | 82168 | 952329 | 0.0001 | 21.1800 | 1 | 10495 | 0.0012 | 12.7726 | 100.0000 | 72.5722 |
| stanford | 281903 | 2594400 | 0.0000 | 16.4063 | 21410 | 2403 | 7.5948 | 0.8524 | 100.0000 | 16.0522 |
| vote | 7115 | 110804 | 0.0022 | 29.1466 | 4734 | 1005 | 66.5355 | 14.1251 | 100.0000 | 2.9049 |
| ythan | 135 | 732 | 0.0402 | 8.8444 | 1 | 52 | 0.7407 | 38.5185 | 100.0000 | 0.1678 |
| beam | 348 | 60900 | 0.5029 | 348.0000 | 1 | 1 | 0.2874 | 0.2874 | 100.0000 | 33.5893 |
| build | 48 | 1200 | 0.5208 | 48.0000 | 1 | 1 | 2.0833 | 2.0833 | 100.0000 | 35.2113 |
| CDplayer | 120 | 240 | 0.0167 | 2.0000 | 60 | 60 | 50.0000 | 50.0000 | 100.0000 | 100.0000 |
| eady | 598 | 357406 | 0.9994 | 1193.3378 | 1 | 1 | 0.1672 | 0.1672 | 100.0000 | 99.8924 |
| fom | 1006 | 1012 | 0.0010 | 0.0119 | 1003 | 1003 | 99.7018 | 99.7018 | 100.0000 | 100.0000 |
| heatCont | 200 | 598 | 0.0150 | 3.9800 | 1 | 1 | 0.5000 | 0.5000 | 100.0000 | 100.0000 |
| heatDisc | 200 | 598 | 0.0150 | 3.9800 | 1 | 1 | 0.5000 | 0.5000 | 100.0000 | 100.0000 |
| iss | 270 | 540 | 0.0074 | 2.0000 | 135 | 135 | 50.0000 | 50.0000 | 100.0000 | 100.0000 |
| MNA_1 | 578 | 1928 | 0.0058 | 4.6713 | 3 | 3 | 0.5190 | 0.5190 | 100.0000 | 100.0000 |
| MNA_2 | 9223 | 30601 | 0.0004 | 4.6358 | 4 | 4 | 0.0434 | 0.0434 | 100.0000 | 100.0000 |
| MNA_3 | 4863 | 15833 | 0.0007 | 4.5116 | 9 | 9 | 0.1851 | 0.1851 | 100.0000 | 100.0000 |
| MNA_4 | 980 | 3256 | 0.0034 | 4.6449 | 2 | 2 | 0.2041 | 0.2041 | 100.0000 | 100.0000 |
| MNA_5 | 10913 | 54247 | 0.0005 | 7.9417 | 10 | 10 | 0.0916 | 0.0916 | 100.0000 | 100.0000 |
| orrSom | 100 | 10000 | 1.0000 | 198.0000 | 1 | 1 | 1.0000 | 1.0000 | 100.0000 | 100.0000 |
| pde | 84 | 382 | 0.0541 | 7.0952 | 1 | 1 | 1.1905 | 1.1905 | 100.0000 | 100.0000 |
| peec | 480 | 1522 | 0.0066 | 4.3417 | 1 | 1 | 0.2083 | 0.2083 | 100.0000 | 100.0000 |
| random | 200 | 2132 | 0.0533 | 19.3200 | 1 | 1 | 0.5000 | 0.5000 | 100.0000 | 2.1682 |
| tline | 256 | 256 | 0.0039 | 0.0000 | 256 | 256 | 100.0000 | 100.0000 | 100.0000 | 0.0000 |

Table S4: **Results of examinations of networks with interaction dynamic.** In header, $N$ yields the number of nodes, $|E|$ yields the number of edges, $D$ yields the density, $< k >$ yields the average degree, $N_D$ yields the number of driver nodes, $N_S$ yields the number of sensor nodes, $\%_D$ yields the proportion of driver and all nodes, $\%_S$ yields the proportion of sensor and all nodes, $\%_{si}$ yields the percentage of self-influencing, and $\%_{int}$ yields the percentage of interactions.

| Network | $N$ | $\|E\|$ | $D$ | $< k >$ | $N_D$ | $N_S$ | $\%_D$ | $\%_S$ | $\%_{si}$ | $\%_{int}$ |
|---|---|---|---|---|---|---|---|---|---|---|
| celegans | 297 | 4296 | 0.0487 | 28.9293 | 14 | 14 | 4.7138 | 4.7138 | 0.0000 | 100.0000 |
| ecoli | 418 | 1038 | 0.0059 | 4.9665 | 233 | 233 | 55.7416 | 55.7416 | 0.0000 | 100.0000 |
| mac71 | 71 | 876 | 0.1738 | 24.6761 | 1 | 1 | 1.4085 | 1.4085 | 0.0000 | 100.0000 |
| mac95 | 94 | 3030 | 0.3429 | 64.4681 | 3 | 3 | 3.1915 | 3.1915 | 0.0000 | 100.0000 |
| s208 | 122 | 378 | 0.0254 | 6.1967 | 1 | 1 | 0.8197 | 0.8197 | 0.0000 | 100.0000 |
| s420 | 252 | 798 | 0.0126 | 6.3333 | 3 | 3 | 1.1905 | 1.1905 | 0.0000 | 100.0000 |
| s838 | 512 | 1638 | 0.0062 | 6.3984 | 11 | 11 | 2.1484 | 2.1484 | 0.0000 | 100.0000 |
| yeast | 688 | 2156 | 0.0046 | 6.2674 | 450 | 450 | 65.4070 | 65.4070 | 0.0000 | 100.0000 |
| amazon0302 | 262111 | 1799584 | 0.0000 | 13.7315 | 442 | 442 | 0.1686 | 0.1686 | 0.0000 | 100.0000 |
| amazon0312 | 400727 | 4699738 | 0.0000 | 23.4561 | 2533 | 2533 | 0.6321 | 0.6321 | 0.0000 | 100.0000 |
| amazon0505 | 410236 | 4878874 | 0.0000 | 23.7857 | 2658 | 2658 | 0.6479 | 0.6479 | 0.0000 | 100.0000 |
| amazon0601 | 403394 | 4886816 | 0.0000 | 24.2285 | 823 | 823 | 0.2040 | 0.2040 | 0.0000 | 100.0000 |
| berkstan | 685230 | 13298940 | 0.0000 | 38.8160 | 191369 | 191369 | 27.9277 | 27.9277 | 0.0000 | 100.0000 |
| caida | 26475 | 106762 | 0.0002 | 8.0651 | 19112 | 19112 | 72.1889 | 72.1889 | 0.0000 | 100.0000 |
| dolphins | 62 | 318 | 0.0827 | 10.2581 | 2 | 2 | 3.2258 | 3.2258 | 0.0000 | 100.0000 |
| epinion | 75879 | 811480 | 0.0001 | 21.3888 | 31720 | 31720 | 41.8034 | 41.8034 | 0.0000 | 100.0000 |
| freeman2 | 34 | 948 | 0.8201 | 55.7647 | 1 | 1 | 2.9412 | 2.9412 | 0.0000 | 100.0000 |
| gnutella04 | 10876 | 79988 | 0.0007 | 14.7091 | 2180 | 2180 | 20.0441 | 20.0441 | 0.0000 | 100.0000 |
| gnutella05 | 8846 | 63678 | 0.0008 | 14.3970 | 1992 | 1992 | 22.5187 | 22.5187 | 0.0000 | 100.0000 |
| gnutella06 | 8717 | 63050 | 0.0008 | 14.4660 | 1907 | 1907 | 21.8768 | 21.8768 | 0.0000 | 100.0000 |
| gnutella08 | 6301 | 41554 | 0.0010 | 13.1897 | 2194 | 2194 | 34.8199 | 34.8199 | 0.0000 | 100.0000 |
| gnutella09 | 8114 | 52026 | 0.0008 | 12.8238 | 2971 | 2971 | 36.6157 | 36.6157 | 0.0000 | 100.0000 |
| gnutella24 | 26518 | 130738 | 0.0002 | 9.8603 | 12111 | 12111 | 45.6709 | 45.6709 | 0.0000 | 100.0000 |
| gnutella25 | 22687 | 109410 | 0.0002 | 9.6452 | 10665 | 10665 | 47.0093 | 47.0093 | 0.0000 | 100.0000 |
| gnutella30 | 36682 | 176656 | 0.0001 | 9.6318 | 18153 | 18153 | 49.4875 | 49.4875 | 0.0000 | 100.0000 |
| gnutella31 | 62586 | 295784 | 0.0001 | 9.4521 | 31209 | 31209 | 49.8658 | 49.8658 | 0.0000 | 100.0000 |
| google | 875713 | 8644102 | 0.0000 | 19.7419 | 268028 | 268028 | 30.6068 | 30.6068 | 0.0000 | 100.0000 |
| grass | 88 | 274 | 0.0354 | 6.2273 | 22 | 22 | 25.0000 | 25.0000 | 0.0000 | 100.0000 |
| notre | 325729 | 2207671 | 0.0000 | 13.3867 | 188276 | 188276 | 57.8014 | 57.8014 | 8.4288 | 100.0000 |
| pokec | 1632803 | 44603928 | 0.0000 | 54.6348 | 70671 | 70671 | 4.3282 | 4.3282 | 0.0000 | 100.0000 |
| slash08 | 77360 | 1015667 | 0.0002 | 24.2596 | 5 | 5 | 0.0065 | 0.0065 | 99.9315 | 100.0000 |
| slash09 | 82168 | 1086763 | 0.0002 | 24.5463 | 71 | 71 | 0.0864 | 0.0864 | 95.2962 | 100.0000 |
| stanford | 281903 | 3985272 | 0.0001 | 28.2741 | 64488 | 64488 | 22.8760 | 22.8760 | 0.0000 | 100.0000 |
| vote | 7115 | 201524 | 0.0040 | 56.6476 | 2637 | 2637 | 37.0625 | 37.0625 | 0.0000 | 100.0000 |
| ythan | 135 | 1196 | 0.0656 | 17.6593 | 23 | 23 | 17.0370 | 17.0370 | 2.9630 | 100.0000 |
| beam | 348 | 90828 | 0.7500 | 521.0000 | 1 | 1 | 0.2874 | 0.2874 | 50.0000 | 100.0000 |
| build | 48 | 1728 | 0.7500 | 71.0000 | 1 | 1 | 2.0833 | 2.0833 | 50.0000 | 100.0000 |
| CDplayer | 120 | 240 | 0.0167 | 2.0000 | 60 | 60 | 50.0000 | 50.0000 | 100.0000 | 100.0000 |
| eady | 598 | 357598 | 1.0000 | 1193.9799 | 1 | 1 | 0.1672 | 0.1672 | 100.0000 | 100.0000 |
| fom | 1006 | 1012 | 0.0010 | 0.0119 | 1003 | 1003 | 99.7018 | 99.7018 | 100.0000 | 100.0000 |
| heatCont | 200 | 598 | 0.0150 | 3.9800 | 1 | 1 | 0.5000 | 0.5000 | 100.0000 | 100.0000 |
| heatDisc | 200 | 598 | 0.0150 | 3.9800 | 1 | 1 | 0.5000 | 0.5000 | 100.0000 | 100.0000 |
| iss | 270 | 405 | 0.0056 | 2.0000 | 135 | 135 | 50.0000 | 50.0000 | 50.0000 | 100.0000 |
| MNA_1 | 578 | 1694 | 0.0051 | 4.6713 | 3 | 3 | 0.5190 | 0.5190 | 59.5156 | 100.0000 |
| MNA_2 | 9223 | 27003 | 0.0003 | 4.6358 | 4 | 4 | 0.0434 | 0.0434 | 60.9888 | 100.0000 |
| MNA_3 | 4863 | 13921 | 0.0006 | 4.5116 | 9 | 9 | 0.1851 | 0.1851 | 60.6827 | 100.0000 |
| MNA_4 | 980 | 2872 | 0.0030 | 4.6449 | 2 | 2 | 0.2041 | 0.2041 | 60.8163 | 100.0000 |
| MNA_5 | 10913 | 54159 | 0.0005 | 7.9417 | 10 | 10 | 0.0916 | 0.0916 | 99.1936 | 100.0000 |
| orrSom | 100 | 10000 | 1.0000 | 198.0000 | 1 | 1 | 1.0000 | 1.0000 | 100.0000 | 100.0000 |
| pde | 84 | 382 | 0.0541 | 7.0952 | 1 | 1 | 1.1905 | 1.1905 | 100.0000 | 100.0000 |
| peec | 480 | 1346 | 0.0058 | 4.3417 | 1 | 1 | 0.2083 | 0.2083 | 63.3333 | 100.0000 |
| random | 200 | 3982 | 0.0996 | 37.8200 | 1 | 1 | 0.5000 | 0.5000 | 100.0000 | 100.0000 |
| tline | 256 | 256 | 0.0039 | 0.0000 | 256 | 256 | 100.0000 | 100.0000 | 100.0000 | 0.0000 |

Table S5: **Results of examinations of networks with self-influence type connections.** $N$ the number of nodes, $|E|$ the number of edges, $D$ the density, $< k >$ the average degree, $N_D$ the number of driver nodes, $N_S$ the number of sensor nodes, $\%_D$ the proportion of driver and all nodes, $\%_S$ the proportion of sensor and all nodes, $\%_{si}$ the percentage of self-influencing connections, and $\%_{int}$ the percentage of interactions.

| Network | $N$ | $\|E\|$ | $D$ | $< k >$ | $N_D$ | $N_S$ | $\%_D$ | $\%_S$ | $\%_{si}$ | $\%_{int}$ |
|---|---|---|---|---|---|---|---|---|---|---|
| celegans | 297 | 4593 | 0.0521 | 28.9293 | 1 | 1 | 0.3367 | 0.3367 | 100.0000 | 100.0000 |
| ecoli | 418 | 1456 | 0.0083 | 4.9665 | 29 | 29 | 6.9378 | 6.9378 | 100.0000 | 100.0000 |
| mac71 | 71 | 947 | 0.1879 | 24.6761 | 1 | 1 | 1.4085 | 1.4085 | 100.0000 | 100.0000 |
| mac95 | 94 | 3124 | 0.3536 | 64.4681 | 1 | 1 | 1.0638 | 1.0638 | 100.0000 | 100.0000 |
| s208 | 122 | 500 | 0.0336 | 6.1967 | 1 | 1 | 0.8197 | 0.8197 | 100.0000 | 100.0000 |
| s420 | 252 | 1050 | 0.0165 | 6.3333 | 1 | 1 | 0.3968 | 0.3968 | 100.0000 | 100.0000 |
| s838 | 512 | 2150 | 0.0082 | 6.3984 | 1 | 1 | 0.1953 | 0.1953 | 100.0000 | 100.0000 |
| yeast | 688 | 2844 | 0.0060 | 6.2674 | 11 | 11 | 1.5988 | 1.5988 | 100.0000 | 100.0000 |
| amazon0302 | 262111 | 2061695 | 0.0000 | 13.7315 | 1 | 1 | 0.0004 | 0.0004 | 100.0000 | 100.0000 |
| amazon0312 | 400727 | 5100465 | 0.0000 | 23.4561 | 1 | 1 | 0.0002 | 0.0002 | 100.0000 | 100.0000 |
| amazon0505 | 410236 | 5289110 | 0.0000 | 23.7857 | 1 | 1 | 0.0002 | 0.0002 | 100.0000 | 100.0000 |
| amazon0601 | 403394 | 5290210 | 0.0000 | 24.2285 | 7 | 7 | 0.0017 | 0.0017 | 100.0000 | 100.0000 |
| berkstan | 685230 | 13984170 | 0.0000 | 38.8160 | 676 | 676 | 0.0987 | 0.0987 | 100.0000 | 100.0000 |
| caida | 26475 | 133237 | 0.0002 | 8.0651 | 1 | 1 | 0.0038 | 0.0038 | 100.0000 | 100.0000 |
| dolphins | 62 | 380 | 0.0989 | 10.2581 | 1 | 1 | 1.6129 | 1.6129 | 100.0000 | 100.0000 |
| epinion | 75879 | 887359 | 0.0002 | 21.3888 | 2 | 2 | 0.0026 | 0.0026 | 100.0000 | 100.0000 |
| freeman2 | 34 | 982 | 0.8495 | 55.7647 | 1 | 1 | 2.9412 | 2.9412 | 100.0000 | 100.0000 |
| gnutella04 | 10876 | 90864 | 0.0008 | 14.7091 | 1 | 1 | 0.0092 | 0.0092 | 100.0000 | 100.0000 |
| gnutella05 | 8846 | 72524 | 0.0009 | 14.3970 | 3 | 3 | 0.0339 | 0.0339 | 100.0000 | 100.0000 |
| gnutella06 | 8717 | 71767 | 0.0009 | 14.4660 | 1 | 1 | 0.0115 | 0.0115 | 100.0000 | 100.0000 |
| gnutella08 | 6301 | 47855 | 0.0012 | 13.1897 | 2 | 2 | 0.0317 | 0.0317 | 100.0000 | 100.0000 |
| gnutella09 | 8114 | 60140 | 0.0009 | 12.8238 | 6 | 6 | 0.0739 | 0.0739 | 100.0000 | 100.0000 |
| gnutella24 | 26518 | 157256 | 0.0002 | 9.8603 | 11 | 11 | 0.0415 | 0.0415 | 100.0000 | 100.0000 |
| gnutella25 | 22687 | 132097 | 0.0003 | 9.6452 | 13 | 13 | 0.0573 | 0.0573 | 100.0000 | 100.0000 |
| gnutella30 | 36682 | 213338 | 0.0002 | 9.6318 | 12 | 12 | 0.0327 | 0.0327 | 100.0000 | 100.0000 |
| gnutella31 | 62586 | 358370 | 0.0001 | 9.4521 | 12 | 12 | 0.0192 | 0.0192 | 100.0000 | 100.0000 |
| google | 875713 | 9519815 | 0.0000 | 19.7419 | 2746 | 2746 | 0.3136 | 0.3136 | 100.0000 | 100.0000 |
| grass | 88 | 362 | 0.0467 | 6.2273 | 1 | 1 | 1.1364 | 1.1364 | 100.0000 | 100.0000 |
| notre | 325729 | 2505945 | 0.0000 | 13.3867 | 1 | 1 | 0.0003 | 0.0003 | 100.0000 | 100.0000 |
| pokec | 1632803 | 46236731 | 0.0000 | 54.6348 | 1 | 1 | 0.0001 | 0.0001 | 100.0000 | 100.0000 |
| slash08 | 77360 | 1015720 | 0.0002 | 24.2596 | 1 | 1 | 0.0013 | 0.0013 | 100.0000 | 100.0000 |
| slash09 | 82168 | 1090628 | 0.0002 | 24.5463 | 1 | 1 | 0.0012 | 0.0012 | 100.0000 | 100.0000 |
| stanford | 281903 | 4267175 | 0.0001 | 28.2741 | 365 | 365 | 0.1295 | 0.1295 | 100.0000 | 100.0000 |
| vote | 7115 | 208639 | 0.0041 | 56.6476 | 24 | 24 | 0.3373 | 0.3373 | 100.0000 | 100.0000 |
| ythan | 135 | 1327 | 0.0728 | 17.6593 | 1 | 1 | 0.7407 | 0.7407 | 100.0000 | 100.0000 |
| beam | 348 | 91002 | 0.7514 | 521.0000 | 1 | 1 | 0.2874 | 0.2874 | 100.0000 | 100.0000 |
| build | 48 | 1752 | 0.7604 | 71.0000 | 1 | 1 | 2.0833 | 2.0833 | 100.0000 | 100.0000 |
| CDplayer | 120 | 240 | 0.0167 | 2.0000 | 60 | 60 | 50.0000 | 50.0000 | 100.0000 | 100.0000 |
| eady | 598 | 357598 | 1.0000 | 1193.9799 | 1 | 1 | 0.1672 | 0.1672 | 100.0000 | 100.0000 |
| fom | 1006 | 1012 | 0.0010 | 0.0119 | 1003 | 1003 | 99.7018 | 99.7018 | 100.0000 | 100.0000 |
| heatCont | 200 | 598 | 0.0150 | 3.9800 | 1 | 1 | 0.5000 | 0.5000 | 100.0000 | 100.0000 |
| heatDisc | 200 | 598 | 0.0150 | 3.9800 | 1 | 1 | 0.5000 | 0.5000 | 100.0000 | 100.0000 |
| iss | 270 | 540 | 0.0074 | 2.0000 | 135 | 135 | 50.0000 | 50.0000 | 100.0000 | 100.0000 |
| MNA_1 | 578 | 1928 | 0.0058 | 4.6713 | 3 | 3 | 0.5190 | 0.5190 | 100.0000 | 100.0000 |
| MNA_2 | 9223 | 30601 | 0.0004 | 4.6358 | 4 | 4 | 0.0434 | 0.0434 | 100.0000 | 100.0000 |
| MNA_3 | 4863 | 15833 | 0.0007 | 4.5116 | 9 | 9 | 0.1851 | 0.1851 | 100.0000 | 100.0000 |
| MNA_4 | 980 | 3256 | 0.0034 | 4.6449 | 2 | 2 | 0.2041 | 0.2041 | 100.0000 | 100.0000 |
| MNA_5 | 10913 | 54247 | 0.0005 | 7.9417 | 10 | 10 | 0.0916 | 0.0916 | 100.0000 | 100.0000 |
| orrSom | 100 | 10000 | 1.0000 | 198.0000 | 1 | 1 | 1.0000 | 1.0000 | 100.0000 | 100.0000 |
| pde | 84 | 382 | 0.0541 | 7.0952 | 1 | 1 | 1.1905 | 1.1905 | 100.0000 | 100.0000 |
| peec | 480 | 1522 | 0.0066 | 4.3417 | 1 | 1 | 0.2083 | 0.2083 | 100.0000 | 100.0000 |
| random | 200 | 3982 | 0.0996 | 37.8200 | 1 | 1 | 0.5000 | 0.5000 | 100.0000 | 100.0000 |
| tline | 256 | 256 | 0.0039 | 0.0000 | 256 | 256 | 100.0000 | 100.0000 | 100.0000 | 0.0000 |

## References


[1] Liu, Y.-Y., Slotine, J.-J. & Barabási, A.-L. Observability of complex systems. *Proceedings of the National Academy of Sciences* **110**, 2460–2465 (2013).

[2] Chahlaoui, Y. & Sima, V. Benchmark Examples for Model Reduction. `http://slicot.org/20-site/126-benchmark-examples-for-model-reduction` (2006). [Online; accessed 04-October-2016].

[3] Chahlaoui, Y. & Van Dooren, P. A collection of benchmark examples for model reduction of linear time invariant dynamical systems. (2002).

[4] Abonyi, J. MATLAB Programs - Data Mining and Complex Systems Laboratory. `http://www.abonyilab.com/software-and-data` (2016). [Online; accessed 04-October-2016].

[5] Watts, D. J. & Strogatz, S. H. Collective dynamics of 'small-world' networks. *nature* **393**, 440–442 (1998).

[6] Shen-Orr, S. S., Milo, R., Mangan, S. & Alon, U. Network motifs in the transcriptional regulation network of escherichia coli. *Nature genetics* **31**, 64–68 (2002).

[7] Young, M. P. The organization of neural systems in the primate cerebral cortex. *Proceedings of the Royal Society of London B: Biological Sciences* **252**, 13–18 (1993).

[8] Kaiser, M. & Hilgetag, C. C. Nonoptimal component placement, but short processing paths, due to long-distance projections in neural systems. *PLoS Comput Biol* **2**, e95 (2006).

[9] Milo, R. *et al.* Superfamilies of evolved and designed networks. *Science* **303**, 1538–1542 (2004).

[10] Milo, R. *et al.* Network motifs: simple building blocks of complex networks. *Science* **298**, 824–827 (2002).

[11] Leskovec, J., Adamic, L. A. & Huberman, B. A. The dynamics of viral marketing. *ACM Transactions on the Web (TWEB)* **1**, 5 (2007a).

[12] Leskovec, J., Kleinberg, J. & Faloutsos, C. Graphs over time: densification laws, shrinking diameters and possible explanations. In *Proceedings of the eleventh ACM SIGKDD international conference on Knowledge discovery in data mining*, 177–187 (ACM, 2005).

[13] Leskovec, J., Lang, K. J., Dasgupta, A. & Mahoney, M. W. Community structure in large networks: Natural cluster sizes and the absence of large well-defined clusters. *Internet Mathematics* **6**, 29–123 (2009).

[14] Lusseau, D. *et al.* The bottlenose dolphin community of doubtful sound features a large proportion of long-lasting associations. *Behavioral Ecology and Sociobiology* **54**, 396–405 (2003).

[15] Richardson, M., Agrawal, R. & Domingos, P. Trust management for the semantic web. In *The Semantic Web-ISWC 2003*, 351–368 (Springer, 2003).

[16] Freeman, S. & Freeman, L. *The Networkers Network: A Study of the Impact of a New Communications Medium on Sociometric Structure.* Social sciences research reports (School of Social Sciences University of Calif., 1979). URL `https://books.google.hu/books?id=sN9NGwAACAAJ`.

[17] Leskovec, J., Kleinberg, J. & Faloutsos, C. Graph evolution: Densification and shrinking diameters. *ACM Transactions on Knowledge Discovery from Data (TKDD)* **1**, 2 (2007b).

[18] Ripeanu, M. & Foster, I. Mapping the gnutella network: Macroscopic properties of large-scale peer-to-peer systems. In *Peer-to-Peer Systems*, 85–93 (Springer, 2002).

[19] Dunne, J. A., Williams, R. J. & Martinez, N. D. Food-web structure and network theory: the role of connectance and size. *Proceedings of the National Academy of Sciences* **99**, 12917–12922 (2002).

[20] Albert, R., Jeong, H. & Barabási, A.-L. Internet: Diameter of the world-wide web. *Nature* **401**, 130–131 (1999).

[21] Takac, L. & Zabovsky, M. Data analysis in public social networks. In *International Scientific Conference and International Workshop Present Day Trends of Innovations*, 1–6 (2012).

[22] Leskovec, J., Huttenlocher, D. & Kleinberg, J. Signed networks in social media. In *Proceedings of the SIGCHI conference on human factors in computing systems*, 1361–1370 (ACM, 2010).

[23] Penzl, T. Algorithms for model reduction of large dynamical systems. *Linear Algebra and its Applications* **415**, 322–343 (2006).
